# EPA and DHA Alleviated Chronic Dextran Sulfate Sodium Exposure-Induced Depressive-like Behaviors in Mice and Potential Mechanisms Involved

**DOI:** 10.3390/md22020076

**Published:** 2024-01-31

**Authors:** Xi-Yu Wang, Shu-Sen He, Miao-Miao Zhou, Xiao-Ran Li, Cheng-Cheng Wang, Ying-Cai Zhao, Chang-Hu Xue, Hong-Xia Che

**Affiliations:** 1 College of Marine Science and Biological Engineering, Shandong Provincial Key Laboratory of Biochemical Engineering, Qingdao University of Science and Technology, Qingdao 266042, China; wangxy99102022@163.com (X.-Y.W.); 15621498160@163.com (S.-S.H.); 15633872044@163.com (M.-M.Z.); 18253813121@163.com (X.-R.L.); 2 SKL of Marine Food Processing & Safety Control, College of Food Science and Engineering, Ocean University of China, No. 1299 Sansha Road, Qingdao 266404, China; wangchengcheng@ouc.edu.cn (C.-C.W.); zhao764089350@163.com (Y.-C.Z.)

**Keywords:** colitis induced depression, EPA, DHA, inflammation, gut microbiota

## Abstract

Patients with ulcerative colitis (UC) have higher rates of depression. However, the mechanism of depression development remains unclear. The improvements of EPA and DHA on dextran sulfate sodium (DSS)-induced UC have been verified. Therefore, the present study mainly focused on the effects of EPA and DHA on UC-induced depression in C57BL/6 mice and the possible mechanisms involved. A forced swimming test and tail suspension experiment showed that EPA and DHA significantly improved DSS-induced depressive-like behavior. Further analysis demonstrated that EPA and DHA could significantly suppress the inflammation response of the gut and brain by regulating the NLRP3/ASC signal pathway. Moreover, intestine and brain barriers were maintained by enhancing ZO-1 and occludin expression. In addition, EPA and DHA also increased the serotonin (5-HT) concentration and synaptic proteins. Interestingly, EPA and DHA treatments increased the proportion of dominant bacteria, alpha diversity, and beta diversity. In conclusion, oral administration of EPA and DHA alleviated UC-induced depressive-like behavior in mice by modulating the inflammation, maintaining the mucosal and brain barriers, suppressing neuronal damage and reverting microbiota changes.

## 1. Introduction

Ulcerative colitis (UC) is characterized by chronic relapsing inflammation affecting the rectum and colon. Common symptoms of UC include weight loss, frequent abdominal pain, diarrhea, and blood in the stools, which significantly affect patients’ quality of life. The global prevalence and incidence of UC have been increasing in recent years, making it a significant public health concern [1]. Patients with UC not only suffer from abdominal pain and diarrhea, but may also experience the torment of depression. Accumulated research has demonstrated that people with UC have a higher tendency of neuropsychiatric disorders such as depression-like phenotypes [2,3]. Repeated treatment of drinking water containing dextran sulfate sodium (DSS) is a classic rodent UC modeling method [4]. Researchers found that mice with DSS administration showed depression and anxiety-like behavior [5,6].

DSS mainly damages the intestinal homeostasis, and its toxicity is manifested in various aspects, including exacerbating apoptosis of intestinal epithelial cells, injuring the integrity of the intestinal epithelial barrier, exacerbating intestinal inflammation, and disrupting the balance of intestinal microbiota [7,8,9]. High molecular weight DSS cannot penetrate the blood–brain barrier (BBB) and directly affects brain tissue homeostasis. However, recent studies have found that DSS-induced colitis mice showed neuroinflammation in the brain and the reduction of neurogenesis [10,11]. Takahashi found a decreased concentration of serotonin, damage of the myelination, increased tumor necrosis factor-α (TNF-α) and interleukin-6 (IL-6), and depressive-like behavior in DSS-induced colitis [12]. Komoto also revealed that C57BL/6J mice treatment with DSS exhibited the high comorbidity of chronic unpredictable mild stress, leading to the induction of depressive disorders [13]. The potential mechanism underlying the connection between intestinal inflammation and depressive symptoms is still unknown. 

The gut microbiota and its metabolites are implicated in the development of inflammatory bowel disease. Compared with the healthy population, the overall diversity of gut microbiota in patients with UC is significantly reduced, with a decrease in the abundance of *Bacteroidetes*, *Firmicutes*, *Clostridium IV* and *Suterella*, and an increase in *Proteobacteria*, *Ruminococcus*, and *Bifidobacterium* [14]. Transplanting healthy gut microbiota into patients with UC through fecal microbiota transplantation could reconstruct normal gut microbiota and achieve the goal of treating diseases [15]. Mounting evidence has suggested that gut microbiota may affect brain function and behavior in the treatment of psychiatric pathology [16,17]. DSS-treated rodents also showed changes of the metabolism of gut microbiota in the intestine, such as the displacement of lipopolysaccharide (LPS), which led to the increase of intestinal permeability (intestinal leakage) and systemic inflammation [18,19]. The systemic inflammatory factors activated the glial cell. The occurrence of inflammation in the brain could be observed in UC-induced depression mice [20,21]. The above research suggests that reshaping the gut microbiota and intestinal inflammatory response may be the key point to improve depression-like behavior caused by UC.

The protective effects of n-3 long-chain polyunsaturated fatty acids docosahexaenoic acid/eicosapentaenoic acid(DHA/EPA) on UC have been extensively reported [22,23]. Previous study has found that dietary supplementation with DHA/EPA could significantly improve DSS-induced colitis by regulating the intestinal barrier, suppressing intestinal inflammation and reshaping the gut microbiota [24,25]. However, the impact of DHA/EPA on colitis-induced depression remains unexplored. The above research revealed that the change in the brain might be due, at least in part, to the altered characteristics of the gut microbiota. Therefore, in the present study, we examined whether DHA/EPA prevented DSS-induced depressive-like behavior in mice, and further explored potential mechanisms from the perspectives of changes in gut microbiota, intestinal inflammation, and intestinal barrier to provide directions for elucidating the mechanism of enteritis leading to psychological symptoms.

## 2. Results

### 2.1. EPA and DHA Alleviated Depressive-like Behavior

**Figure 1:**
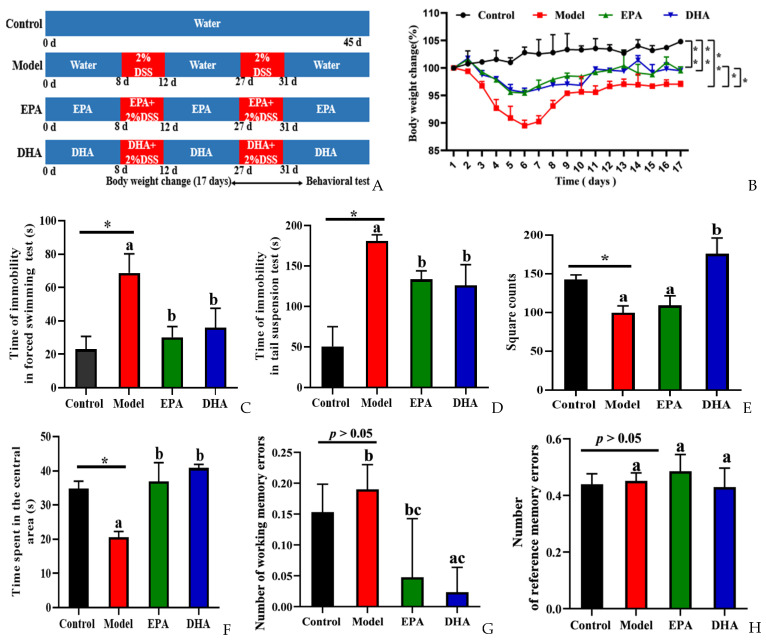
EPA and DHA alleviated DSS-induced depressive-like behavior of C57BL/6 mice. (**A**) Experimental design and timeline of DSS treatment. (**B**) The body weight changes were detected during the second DSS treatment and 12 days after DSS. (**C**,**D**) Time of immobility in the FST and TST. (**E**,**F**) The duration of time the mouse spent in the central area and the total number of grid crossings were recorded in the OFT. (**G**,**H**) Number of working memory errors and reference memory errors in EAM. * *p* < 0.05, ** *p* < 0.01 and different letters are used to indicate statistical differences.

To explore the effects of DSS on depression, we focused on body weight changes, and then behavior experiments, namely a forced swimming test (FST), tail suspension test (TST), open field test (OFT), and the eight-arm maze (EAM), were conducted to evaluate the behavioral characteristics of control and model mice and those supplemented with DHA and EPA for comparison (Figure 1B–H). We recorded the changes of body weight of the mice during the 17 days after starting the second round of DSS treatment. It could be clearly observed that the body weight decreased sharply after five consecutive days of DSS intervention, and then reached its lowest point on the first day after the second round of DSS treatment. However, the decreasing trend was relieved after EPA and DHA supplementation (Figure 1B). 

Decreased desire for survival, lack of exploration of new spaces, and memory loss are all signs of depression-like behavior of mice. Results of FST and TST showed the duration of immobility of the mice in model group was longer than that in the control group. After the intervention with DHA and EPA, the immobility time was equally reduced (Figure 1C,D). The results of OFT showed that the DSS treatment obviously decreased the square counts, which are the total number of grid crossings of the mice. EPA supplementation increased the square counts, but with no statistical significance. Interestingly, DHA obviously increased the square counts. Results of time spent in the center showed that the mice of the model group spent less time in that area (Figure 1E). EPA and DHA supplemented mice spent a longer time in the central area with similar improvement (Figure 1F). The results of EAM showed that the number of working memory errors and reference memory errors of mice in the model group were similar to that of the control group (Figure 1G,H). Supplementation with EPA and DHA decreased the number of working memory errors, and DHA exerted a significant advantage. No significant difference was observed after EPA treatment in decreasing the number of working memory errors (Figure 1G). However, the reference memory errors in the model group and control group were not significantly different, and EPA and DHA treatment showed no advantage (Figure 1H). All of the above results demonstrated that EPA and DHA alleviated depressive-like behavior.

### 2.2. Effects of EPA and DHA on Histological Changes of the Colon

**Figure 2:**
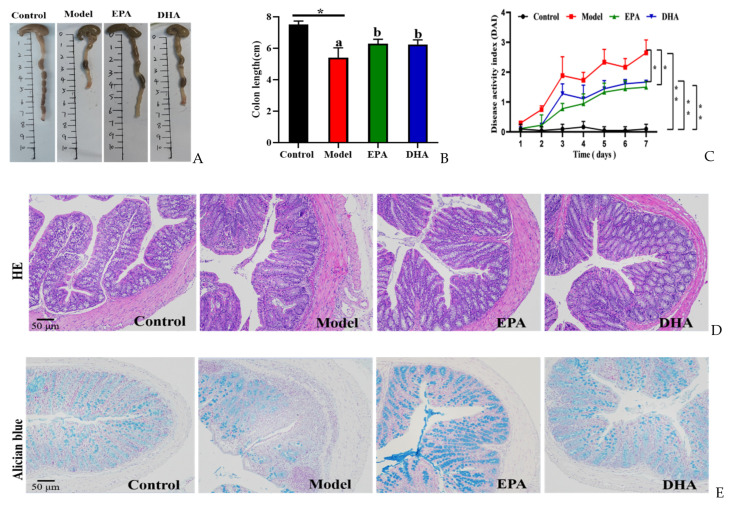

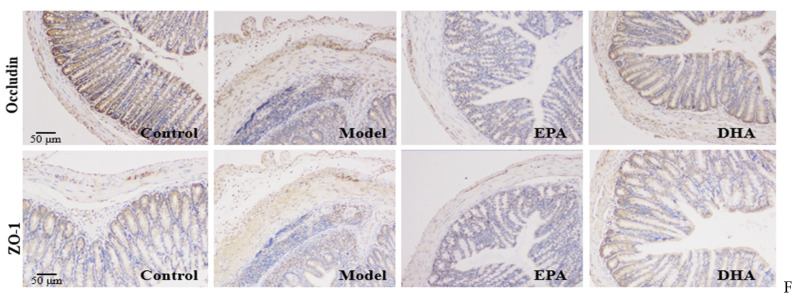
EPA and DHA relieved typical symptoms of UC. (**A**,**B**) Representative images of colon and quantitative analysis of colon length. (**C**) DAI scores. (**D**) Representative results of HE staining (scale bar = 50 μm). (**E**) Alcian blue (scale bar = 50 μm). (**F**) Immunohistochemistry of occludin and ZO-1 in the colon tissue (scale bar = 50 μm). * *p* < 0.05, ** *p* < 0.01 were used to indicate statistical differences of the four groups. * *p* < 0.05, ** *p* < 0.01, and different letters are used to indicate statistical differences.

Compared to the mice of the control group, mice with two rounds of DSS treatment exhibited typical symptoms of colitis, such as reduced colon length (Figure 2A,B) and increased disease activity index (DAI) scores (Figure 2C). Dietary addition of DHA and EPA significantly alleviated the colitis phenotype by inhibiting the colon length shortening and avoiding the elevated DAI scores due to the changes of weight loss, loose stools, and hematochezia in mice. EPA and DHA exerted similar improvement in increasing the colon length and decreasing the DAI score (Figure 2A–C). 

HE staining and Alician blue staining data displayed that an increased intestinal permeability was observed in the model group, such as intestinal structure atrophy (reduced villus height, Figure 2D), and low level acidic mucin level (Figure 2E). At the same time, immunohistochemical data revealed that the expression of tight junction proteins (TJs), including zonula occludens 1 (ZO-1) and occludin, were reduced in the colon of the model mice (Figure 2F). Moreover, long-term EPA/DHA supplementation significantly ameliorated intestinal barrier damage and maintained the integrity of the mechanical barrier of the intestinal mucosa by enhancing the protein expression of ZO-1 and occludin (Figure 2D–F).

### 2.3. EPA and DHA Maintained the Blood–Brain Barrier

**Figure 3:**
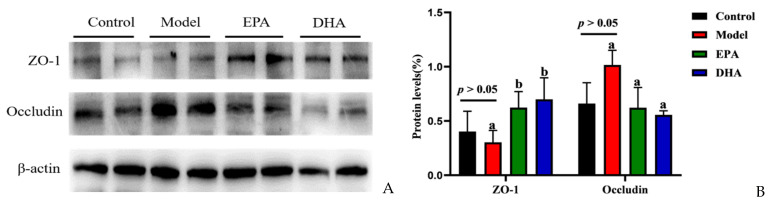
EPA and DHA ameliorated blood–brain barrier. (**A**) The protein expression of ZO-1 and occludin in the brain (**B**) The quantitative analysis of ZO-1 and occludin in the brain. Different letters are used to indicate statistical differences.

Immunohistochemical data have suggested reduced expression of TJs, precisely ZO-1 and occludin, in the intestines of DSS-induced mice (Figure 2F). To investigate the expression of TJs in the brain, we also examined the expression of ZO-1 and occludin by Western blotting (Figure 3A). Unlike changes in intestinal permeability, DSS treatment did not reduce the level of TJs in the brain. The protein expression of ZO-1 was similar between the model group and the control group. However, EPA and DHA supplementation could enhance the expression of the key protein ZO-1 in the brain, but did not increase the level of occludin (Figure 3B). It seemed that the model group showed higher occludin expression. In summary, EPA and DHA may affect the blood–brain barrier by regulating the key protein ZO-1 in TJs.

### 2.4. EPA and DHA Relieved Inflammation in the Intestine and Brain

**Figure 4:**
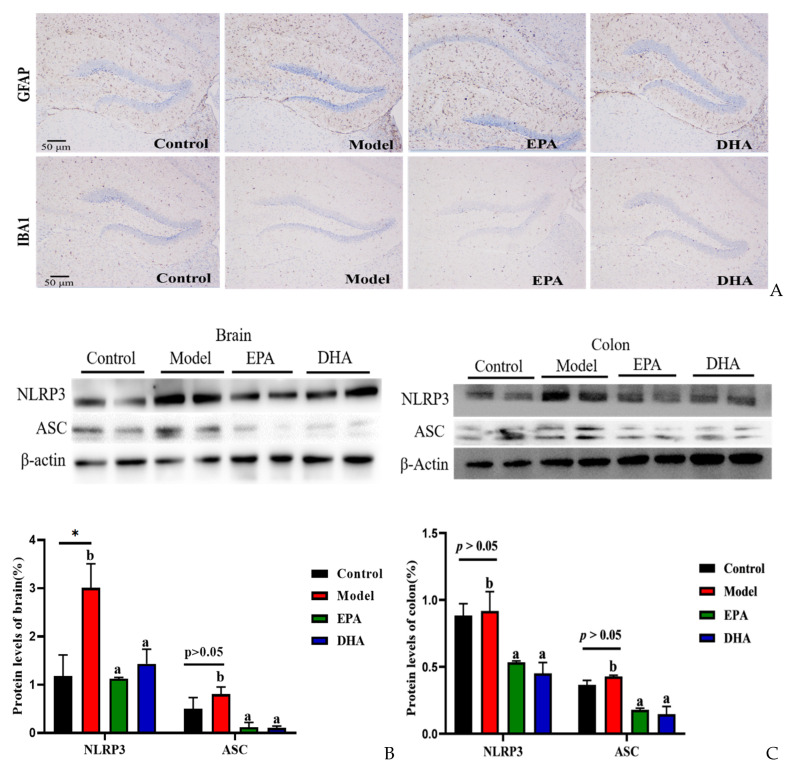

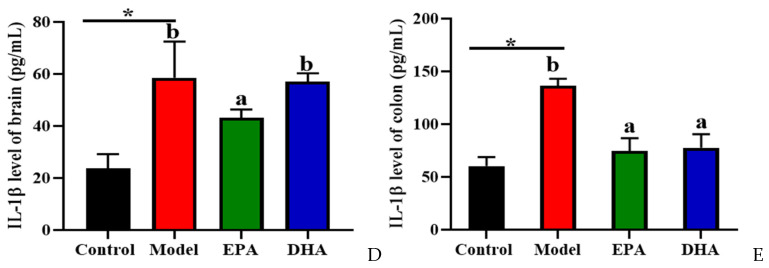
EPA and DHA attenuated inflammation by regulating NLRP3 inflammasome. (**A**) Immunohistochemical representative diagram of activation markers of astrocyte and microglia (scale bar = 50 μm). (**B**,**C**) Key protein expressions of NLRP3 inflammasome and quantitative analysis of the brain and the colon. (**D**,**E**) IL-1β level of the brain and the colon detected by ELISA. * *p* < 0.05 and different letters are used to indicate statistical differences.

The release of inflammatory cytokines is closely related to the activation of cerebral glial cells. Firstly, we focused on the activation of cerebral glial cells and observed the expression of glial fibrillary acidic protein (GFAP) and ionized calcium binding adaptor molecule 1 (IBA-1) by immunohistochemistry. The results showed that the cerebral glial cells of the DSS group were activated, and dietary supplementation with DHA/EPA alleviated this activation (Figure 4A). In addition, we assessed the inflammation in the colon and brain (Figure 4B–E). The results of Western blotting suggested that two rounds of DSS treatment obviously increased the NOD-like receptor thermal protein domain-associated protein 3 (NLRP3) expression both in the brain and the colon. However, as for the apoptosis-associated speck-like protein containing a CARD (ASC) expression, the changes between the model group and the control group were not significant. Supplementation with EPA and DHA obviously decreased the protein expression of NLRP3 and ASC both in the brain and colon of the model group (Figure 4B–C). ELISA results for the cellular inflammatory factor interleukin-1β (IL-1β) showed that the levels of inflammatory factors both in the brain and colon of the model group were almost three times higher than those in the control group. However, EPA treatment significantly decreased the concentration of IL-1β both in the brain and the colon tissue (Figure 4D–E). In contrast, DHA treatment only significantly reduced IL-1β in colon tissue (Fig. 4E), but the effect on brain tissue was not significant (Figure 4D). Overall, EPA was more effective than DHA in regulating the cerebral inflammatory response.

### 2.5. EPA and DHA Suppressed Neuronal Damage

**Figure 5:**
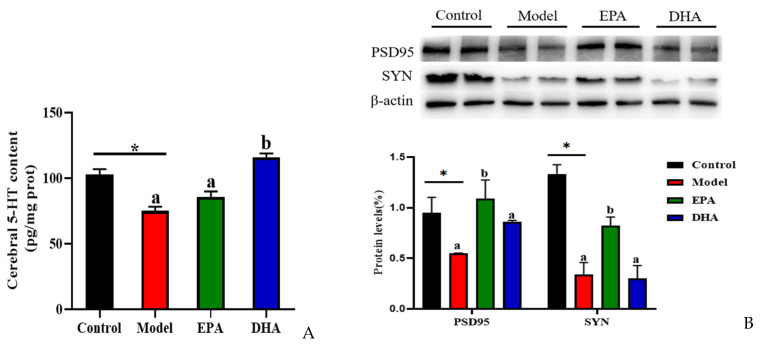
EPA and DHA promoted the expressions of synaptic proteins in the brain of DSS-administered mice. (**A**) Cerebral 5-HT level. (**B**) Representative immunoblots of synaptic proteins and the quantification. * *p* < 0.05 and different letters are used to indicate statistical differences.

The alterations of serotonin (5-HT) and synaptic proteins are critical in the pathogenesis of depression. The concentration of cerebral 5-HT was detected using an ELISA kit. The results indicated that DSS treatment significantly decreased the content of 5-HT in the brain. EPA and DHA supplementation could increase the concentration of 5-HT, and the improvement of DHA was superior to that of EPA. No significant difference was observed between the EPA group and the model group (Figure 5A). Similarly, the results of Western blotting revealed that DSS intervention obviously decreased the synaptic proteins of postsynaptic density protein-95 (PSD95) and synaptophysin (SYN), and EPA-supplemented mice showed higher SYN and PSD95 expression, while DHA had no statistically significant effect (Figure 5B). Therefore, we hypothesized that EPA and DHA ameliorated neuronal injury by increasing the expression of 5-HT, PSD95, and SYN, respectively. 

### 2.6. EPA and DHA Reshaped the Composition of Gut Microbiota

**Figure 6:**
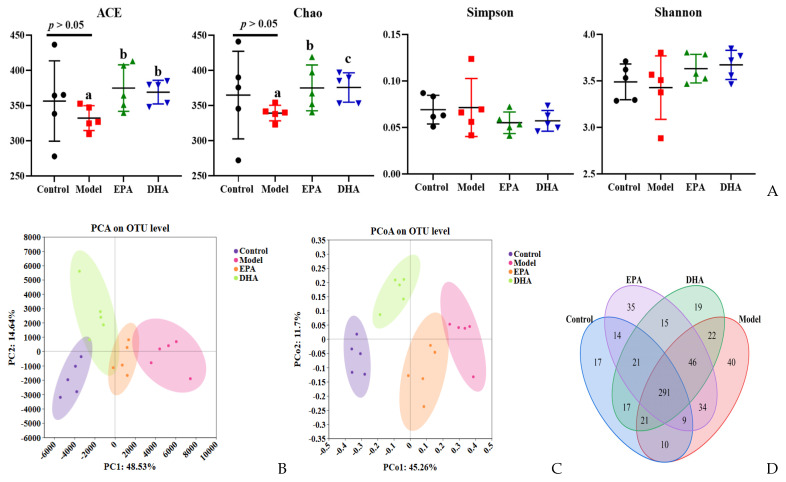

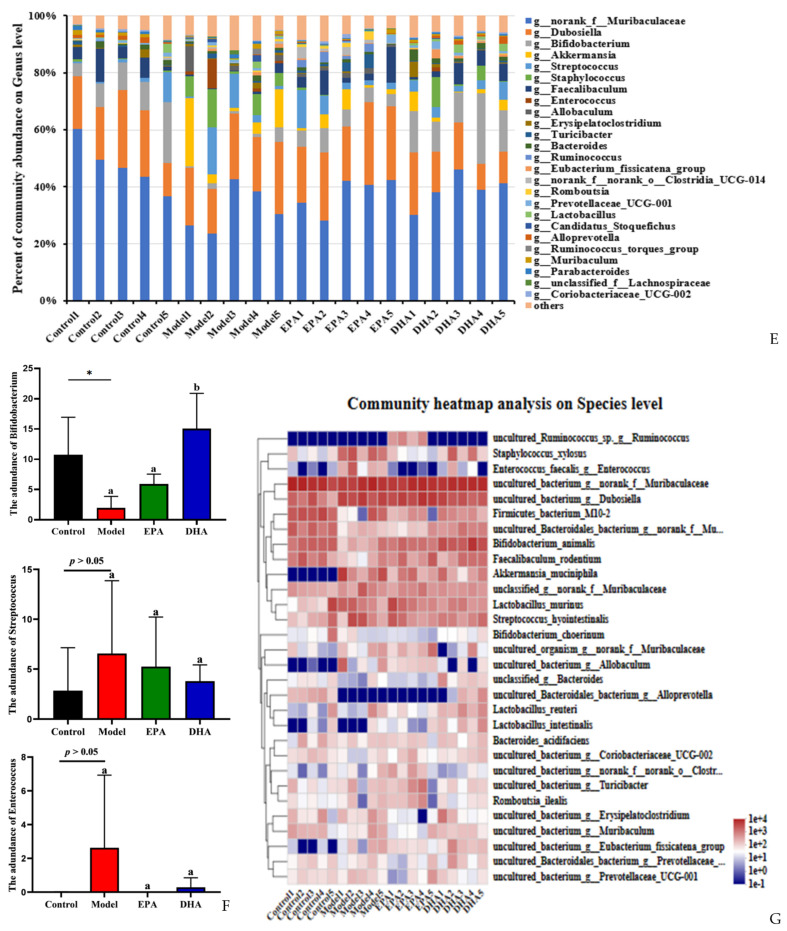
EPA and DHA improved the composition of microbial community structure. (**A**–**C**) Alpha diversity and beta diversity of gut microbiota. (**D**) Venn diagram. (**E**) Distribution of colony abundance at the genus level. (**F**) Changes in *Bifidobacterium*, *Streptococcus*, and *Enterococcus* at genus level. (**G**) Community heat map analysis on species level. * *p* < 0.05 and different letters are used to indicate statistical differences.

DSS intervention induced changes of the diversity and the structural composition of the intestinal flora (Figure 6A–G). As for the changes of flora diversity, DSS exposure decreased the ACE and Chao indexes, and dietary supplementation with DHA/EPA could equally inhibit these decreases (*p* < 0.05) and increase the α-diversity index (Figure 6A). However, the changes of the Simpson and Shannon indices among these four groups were not significant. Furthermore, in the PCA and PCoA plots, the control, model, EPA, and DHA groups showed different clustering of microbial community structure, and the gut microbial structure was changed after DSS, EPA, and DHA intervention (Figure 6B,C). In addition, 611 OTUs were identified in the fecal samples of all four groups as shown in the Venn diagram, of which 291 OTUs were common to all four groups, while 40 OTUs in the model group were different from all other groups (Figure 6D). However, the composition of microbial community structure showed different trends among the four groups (Figure 6E–G).

Next, we determined the composition of the gut microbiota at different taxonomic levels. At genus level, Muribaculaceae, Dubosiella, and Bifidobacterium, approximately accounting for 80%, were the most abundant taxa. Statistical analyses, represented by Bifidobacterium, Streptococcus, and Enterococcus at the genus level, revealed that DSS exposure raised the abundance of Streptococcus and Enterococcus as well as distinctly reducing the abundance of Bifidobacterium. In contrast, the supplementation of EPA and DHA restored the structure of gut microbiota near to that of the control group (Figure 6E–F). At species level, clustered heat maps depicted the abundance of the top 30 species, and the structural composition of the microbial communities of the four groups showed significant differences, especially for *Alloprevotella*, *Allobaculum*, *Akkermansia_muciniphila*, and *Enterococcus* (Figure 6G). These results suggested that DHA and EPA had the ability to enrich the diversity of and reshape the composition of gut microbiota.

## 3. Discussion

In this study, we firstly explored the protective effects of DHA and EPA on DSS-induced depressive-like behavior and cerebral neuronal injury. Our results showed that DSS treatment induced depressive behavior in mice, and glial inflammation and neuronal damage in the brain, and disruption of gut microbiota. Dietary supplementation with DHA and EPA alleviated intestinal and brain inflammation, increased the expression of synaptic related proteins in the brain and regulated gut microbiota. 

Damage to the intestinal barrier, changes in microbiota, and high-level inflammation are typical pathological features of UC [26]. The intestinal mucosal barrier, including mechanical barriers, chemical barriers, immune barriers, biological barriers, is the largest barrier that ensures the homeostasis of the internal environment. The mechanical barrier is composed of TJs, intestinal epithelial cells, and their secreted mucus layer, which is the first line of defense for the body to resist pathogen invasion. Among them, TJs are the main connection between intestinal epithelial cells. ZO-1 and occludin are two classical intercellular tight junction proteins, which are closely related to intestinal integrity. When the components of the mechanical barrier are disrupted, intestinal permeability increases, resulting in bacterial translocation, and exacerbating the progression of UC. Numerous studies have confirmed that DSS could disrupt intestinal mucosal barrier. In our study, we found that DHA and EPA could significantly maintain intestinal mechanical barrier function by enhancing the expression of ZO-1 and occludin, and improve the ability of intestinal epithelial cells to produce mucin, which is consistent with previous studies [24,27]. Our results also suggested that DHA and EPA showed certain advantages in regulating the integrity of the BBB, which is crucial for maintaining brain homeostasis. Increased permeability of the BBB was observed in the brain of patients with depression, manifested by a decrease in TJs’ expression [28]. The above results suggest that the improvement of DHA and EPA in relieving UC-induced depression in mice is at least in part related to the integrity maintenance of intestinal and brain barriers. 

The accumulation of intestinal inflammatory factors, mainly the pro-inflammatory factors, is the main culprit of intestinal barrier damage. It has been revealed that inflammatory factors have a negative impact on intestinal epithelial cells and the intestinal microenvironment, ultimately leading to an unresponsive chronic inflammatory response in the intestine and the development of UC [29,30]. In addition, the leakage of the intestinal barrier can lead to bacterial toxins and pro-inflammatory factors in the intestinal cavity entering the bloodstream, causing systemic inflammatory reactions [31]. Peripheral inflammation is a risk factor for the occurrence of cerebral inflammation and the developing depression [32,33]. Clinical studies found that patients with inflammatory bowel disease had a higher risk of depression [34,35]. Previous study showed that in the acute phase of mouse colitis, the number of macrophages in the brain increased, and at the same time, microglia and the main immune monitoring cells of the nervous system were also activated, indicating that intestinal inflammation can quickly cause brain inflammatory response [36]. We focused on the effects of the NLRP3 inflammasome on peripheral inflammation and brain inflammation, which was the most representative inflammasome responsible for recognizing exogenous and endogenous danger signals [37,38]. Activation of NLRP3 inflammasome promoted the production of proinflammatory cytokines IL-1β. 

Its secretion, in turn, triggered inflammation and participated in the pathogenesis of depression [39,40]. Therefore, the inhibition of IL-1β could serve as an accomplice in the management of UC-associated depression. In the present study, EPA and DHA administration alleviated the damage of DSS treatment on intestinal and cerebral inflammation by downregulating NLRP3 and ASC protein expression, and EPA and DHA exerted similar effects in reducing the production of the pro-inflammatory cytokine IL-1β. Studies have found that people with major depressive disorder showed increased inflammatory cytokines and severe neuroinflammation [41,42]. Our present results showed that EPA was superior to DHA in decreasing the production of cerebral IL-1β. This seems to suggest targeted intervention to reduce intestinal inflammation and related pathological reactions has enormous therapeutic potential in the field of psychiatric disorders. 

DSS-treated mice exhibited dysfunction of the serotonergic system, and reduction of synaptic plasticity markers (SYN and PSD95) in the brain [43,44]. Cerebral serotoninergic systems are subjected to the alterations in the intestinal microenvironment [45]. Serotonin (5-HT) is the most important neurotransmitter in the onset and development of depression. Previous study has reported that both the tryptophan hydroxylase 2, one of the key enzymes for 5-HT synthesis, and 5-HT levels were decreased in the prefrontal cortex in DSS-treated mice [12]. This study also showed that DSS treatment obviously decreased 5-HT levels. Interesting, our results found that DHA and EPA administration obviously relieved the reduction of 5-HT in the brain caused by DSS. In addition, researchers observed a decrease in dendritic complexity and the synaptic number of neurons in the hippocampus of depressed animals. Evidence showed that 5-HT participates in the process of shaping synaptic plasticity [46,47]. Consistent with the changes of 5-HT level, the expression of PSD95 and SYN in the brain showed a decreasing trend after DSS treatment. However, dietary supplementation with EPA and DHA significantly improved synaptic plasticity markers. Therefore, the protective effects of EPA and DHA on depressive-like behavior might partially be associated with suppressing neuronal damage.

Microecological dysbiosis, which is a pathological imbalance in the microbial community, has been linked to UC. Evidence has revealed that the imbalance of intestinal microflora affected the renewal and differentiation of intestinal epithelial cells, the thickness and composition of the mucus layer, the distribution of tight junction proteins, and the microflora metabolites, which may damage the mechanical barrier, causing chronic inflammation of the intestine, leading to the development and progression of ulcerative colitis. The gut microbiome is associated with inflammatory bowel disease and mental health disorders such as anxiety and depression, and increasing evidence has suggested that there are differences in the composition of gut microbiota between healthy individuals and patients with anxiety and depression [48,49]. Numerous studies have shown a decrease in gut microbiota diversity and an increase in the abundance of inflammation-related taxa in UC and depression-related mice models [50,51]. In this study, a chronic enteritis model was established using two rounds of low-dose DSS drinking water. A decreasing trend of α diversity was shown after DSS exposure, but there was no significant difference compared to the control group. This may be caused by our modeling method. Interestingly, intervention with DHA and EPA could significantly improve the diversity index, ACE, and Chao. Moreover, the administration of DHA and EPA affected the abundance of specific bacterial communities. The probiotic *Bifidobacterium* was increased and potential pathogens containing *Streptococcus* and *Enterococcus* were decreased after DHA and EPA supplementation, which were obviously changed in gut microbiota in patients with UC and depression [52,53]. Studies on mice showed that probiotic administration of *Bifidobacterium* seemed to improve UC and depressive symptoms [54,55]. Overall, the potential mechanism by which EPA and DHA improve depression-like behavior caused by UC cannot be separated from the regulation of gut microbiota diversity and composition.

## 4. Materials and Methods

### 4.1. Materials

EPA ethyl ester and DHA ethyl ester (90%purity) were obtained from Xi’an Chiba Grass Biotechnology Co., Ltd (Xi’an, China). DSS (molecular weight 36–50 kDa) used in this study was purchased from MP Biomedicals (Irvine, CA, USA). The primary antibodies of ZO-1 (#GB111402), occludin (#GB111401), and β-actin (#GB15003) were bought from Service-bio (Wuhan, China). NLRP3 (#A5652) was obtained from ABclonal Technology (Wuhan, China). ASC (#67824T) was purchased from Cell Signaling Technology (Boston, MA, USA). SYN (#R25834) and PSD95 (#381001) were purchased from Zen-Bioscience (Chengdu, China). 

### 4.2. Animals and Treatments

Seven- to eight-week-old C57BL/6 mice (*n* = 32, 21–22 g, male) were provided by Jinan Pengyue Experimental Animal Breeding Co., Ltd. (license number: scxk 20190003). All animals were adapted to the breeding environment with a 12 h/12 h light/dark period. The animal experiments were approved by the Animal Ethics Committee of Qingdao University of Science and Technology (no. SYXK2022-0602).

According to our experimental design in Figure 1A, one week later, the mice of the control group and model group were given equal volumes of 0.2% bile salt solution. The EPA group and DHA group were administrated with 100 mg EPA or DHA per kg body weight. In the study, 2 cycles 2% DSS (wt/vol) in drinking water were used to establish a DSS exposure-induced depressive-like behaviors mice model. In the first cycle, mice were given 2% DSS from the 8th day to the 12th day, and then received drinking water without DSS for 2 weeks. Subsequently, mice were treated with 2% DSS from the 27th day to the 31th day followed by 2 weeks distilled water. As shown in Figure 1B, the body weight (%BW) changes were monitored during the second DSS treatment and 12 days after DSS. The DAI score was calculated based on our previous method [56]. 

### 4.3. Behavioral Test

According to our previous study, behavioral tests including FST, TST, OFT, and EAM were conducted one week before the end of the experiment [57]. Specific details of the behavioral tests are included in the supplementary information. Behavioral test data were recorded by the Smart 3.0 software (Panlab, Spain).

### 4.4. Tissue Collection

After the behavioral studies, the mice were fasted for 12 h. The blood was taken from the eyeball and then the mice were sacrificed by cervical dislocation under anesthesia. The colon and brain tissue were quickly collected and stored at −80 °C in a freezer. 

### 4.5. Western Blotting and Other Experiments

Western blotting, ELISA, immunohistochemistry (IHC), HE staining, and Alcian blue staining were conducted according to our previous study; detailed methods can be found in the supplementary information [56].

### 4.6. 16S rRNA Gene Sequencing

Total microbial DNA from the fecal samples was extracted as previously described [58]. The V3-V4 region of the 16S rRNA gene was amplified. The PCR products were purified using Agencourt AMPure XP beads purchased form Beckman Coulter (USA). The sequencing service was completed using an Illumina MiSeq instrument (Illumina, San Diego, CA, USA). Operational taxonomic units (OTUs) with a 97% similarity threshold were clustered by UPARSE software (version 7.1).

### 4.7. Statistical Analyses

Statistical analysis was performed by GraphPad Prism 9. All data were recorded using means ± standard errors. Significant differences were indicated when *p* < 0.05. T-test was used to compare the statistical difference between the control and model groups. Data analyses among the DSS group, DHA group, and EPA group were performed by one-way ANOVA followed by a Tukey’s post hoc test. 

## 5. Conclusions

In the present study, DSS-induced UC model mice initiated depressive-like behavior that might be triggered by the activation of inflammatory response in the intestine and brain tissue, and the imbalance of gut microbiota. In addition, DHA and EPA had preventive effects on these abnormalities potentially through maintaining the gut–brain barrier, inhibiting the activation of the NLRP3 inflammasome pathway in the intestine and brain, and the reconstruction of microbial community structure. This study provided theoretical support for DHA/EPA to ameliorate DSS-induced depression-like behavior in mice, but there were some limitations, such as a single dose level of oral DHA/EPA in mice. However, in the future, we will continue to focus on the effects of different dose relationships of DHA and EPA on the brain’s nervous system, trying to identify the mediators of the communication between the gut and brain. Further studies will be needed to clarify the role of NLRP3 inflammatory and gut microbiota on the pathogenesis of UC-related depression in mice.

## Data Availability

The data presented in this study are available on request from the corresponding author.

## Supplementary Materials

The following supporting information can be downloaded at: https://www.mdpi.com/article/10.3390/md22020076/s1. In the Supplementary file we specifically described behavioral experiments, western blotting, immunohistochemistry, HE staining and Alcian blue staining methods.
