# Supplementary material for: EPA and DHA Alleviated Chronic Dextran Sulfate Sodium Exposure-Induced Depressive-like Behaviors in Mice and Potential Mechanisms Involved
Source: Mar Drugs. 2024 Jan 31;22(2):76. doi: 10.3390/md22020076 (PMC10890276; doi:10.3390/md22020076)
Supplement: Supplementary file 1 [file marinedrugs-22-00076-s001.zip › marinedrugs-2840216-supplementary.pdf]

Supplementary Methods

Behavioral Test

Behavioral tests were conducted one week before the end of the experiment.

Forced swimming test (FST): A transparent cylinder was prepared and filled with water, and the water temperature was maintained at 25°C. The mice were gently placed in the water and forced to swim for 6 min. The time mice spent in the water during the last 4 minutes without struggling and only making the necessary movements to keep their heads above the water was defined as immobility time and recorded using VisuTrack software.

Tail suspension test (TST): The mice's tails were fixed to the crossbar with tape at a distance of approximately 1cm from the tip of the mice's tails. The mice were suspended 20 cm from the ground for 6 min. The time the mice remained stationary for the last 4 min was recorded.

Open Field Test (OFT): The open field is an area (length*width=50*50cm) with 25 (5*5) squares in it. Briefly, the mice were placed into the central grid of the mine field experimental box. The mice were allowed to adapt to the environment for 1 minute. The duration of mouse spent in the central area and the total number of grid crossings (square counts) were recorded. At the end of each test, the open field was wiped with 75% ethanol to eliminate odour disturbance.

The eight-arm maze (EAM): An eight-arm radial maze consisting of eight identical arms extending outwards from the central platform was prepared and the mice were given a strictly controlled diet before the experiment began. The experiment was

conducted over a total of 5 days, with the first two days devoted to environmental adaptation, and each mouse explored freely in each of the eight arms for 10 minutes each day; on the third day, baits were placed in the central area with the end of the arms and the mice were allowed to explore freely for 10 minutes; on the fourth day, four arms were randomly selected, baits were placed at the end and the mice were allowed to explore all the arms; on the fifth day, baits were placed in the same locations and the mice were allowed to explore freely for 10 minutes to see if they remembered the location of the baits. The results of the experiment were recorded as correct for the first entry into the baited arm, as a working memory error for re-entry into the previously entered arm, and as a reference memory error for entry into the unbaited arm.

Western Blotting

The colon and brain tissues of mice frozen at -80°C were homogenized with RIPA lysis buffer containing protease inhibitors. After centrifugation, the supernatant was obtained. The protein concentration was measured using the BCA Protein Concentration Assay Kit (Shanghai Ya Enzyme Biologicals, China) and incubated in boiling water for 10 minutes with denatured protein loading buffer (Shanghai Ya Enzyme Biologicals, China).

The denatured protein was separated by SDS-PAGE and then transferred to PVDF membranes (Wuhan Seville Biologicals, China). The membranes were blocked with 5% BSA (bovine serum albumin) for 2 h at room temperature, incubated with primary antibody at 4 °C overnight and then incubated with horseradish peroxidase-labelled secondary antibody for 2 h at room temperature. Protein expression can be presented

with the aid of an all-in-one chemiluminescence imager (Shanghai Qinxiang Scientific Instruments, China), and quantitative analysis of proteins were processed using Image J software. The primary antibodies involved in this experiment include: β-actin, NLRP3, ASC, ZO-1, occludin, SYN, PSD95.

Immunohistochemistry (IHC)

The colon and brain tissues were fixed overnight in 4% paraformaldehyde, embedded in paraffin and cut into 5-μm tissue sections. Sections were dried at 65°C and treated with 3% hydrogen peroxide after dewax hydration and antigen repair. The tissue sections were closed with 5% BSA for 1 h at room temperature, washed with PBS and incubated overnight at 4°C with the primary antibody. The sections were washed again with PBS, incubated with biotin-coupled secondary antibody for 1 h, DAB for color development and hematoxylin for restaining the nuclei. Finally, the tissue sections were dehydrated and sealed, ready for observation under a light microscope and quantitative analysis using Image J software. The primary antibodies involved in this experiment include: occludin, ZO-1, GFAP, IBA1.

***HE and* Alcian Blue Staining**

The obtained distal colon approximately 1 cm was fixed in 4% paraformaldehyde for 24 h. Paraformaldehyde fixed colon tissue was cut into 5-μm of tissue sections. After dewaxed and hydrated, the nuclei were stained blue-purple with alkaline hematoxylin stain and the cytoplasm was stained red with acidic eosin stain, and the H&E stained sections were sealed by dehydration and ready for observation under a light microscope.

Histopathological scoring of the colon tissue is performed by analyzing the loss of crypt, the degree of epithelial damage and the infiltration of inflammatory cells.

Sections were dewaxed and hydrated and stained with Alcian-Blue for 10 minutes and Nuclear-Fast-Red for 1 minute. After rinsing, the sections were dehydrated in gradient ethanol, clear in xylene and sealed in neutral gum to allow observation of the goblet cells under a light microscope. Quantitative analysis of goblet cells in Alcian Blue stained sections can be performed with the aid of Image J software.
